# Genetic signatures of lineage fusion closely resemble population decline

**DOI:** 10.1002/ece3.10725

**Published:** 2023-11-12

**Authors:** Ryan C. Garrick

**Affiliations:** ^1^ Department of Biology University of Mississippi Oxford Mississippi USA

**Keywords:** Bayesian skyline plot, demographic history, fission‐fusion, neutrality test, phylogeography, population merging

## Abstract

Accurate interpretation of the genetic signatures of past demographic events is crucial for reconstructing evolutionary history. Lineage fusion (complete merging, resulting in a single panmictic population) is a special case of secondary contact that is seldom considered. Here, the circumstances under which lineage fusion can be distinguished from population size constancy, growth, bottleneck, and decline were investigated. Multi‐locus haplotype data were simulated under models of lineage fusion with different divergence versus sampling lag times (D:L ratios). These pseudo‐observed datasets also differed in their allocation of a fixed amount of sequencing resources (number of sampled alleles, haplotype length, number of loci). Distinguishability of lineage fusion versus each of 10 untrue non‐fusion scenarios was quantified based on six summary statistics (neutrality tests). Some datasets were also analyzed using extended Bayesian skyline plots. Results showed that signatures of lineage fusion very closely resemble those of decline—high distinguishability was generally limited to the most favorable scenario (D:L = 9), using the most sensitive summary statistics (*F*_S_ and *Z*_nS_), coupled with the optimal sequencing resource allocation (maximizing number of loci). Also, extended Bayesian skyline plots often erroneously inferred population decline. Awareness of the potential for lineage fusion to carry the hallmarks of population decline is critical.

## Graphical abstract

Statistical phylogeography requires that competing hypotheses make quantitatively different predictions about patterns of genetic variation, and that the true demographic history is represented within the set. Here, simulations showed that signatures of lineage fusion closely resemble population decline. Accordingly, if fusion is not explicitly considered, the risk of false inference becomes elevated.

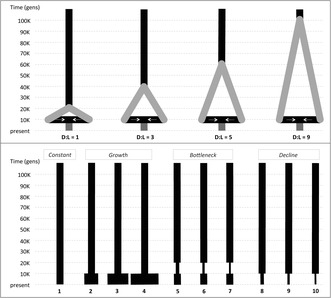

## 1 INTRODUCTION

Lineage fusion (sensu Campbell et al., 2008) is the complete merging of two or more populations resulting in a single panmictic group for which the parental lineages are no longer extant. When considering term “lineage” broadly, this phenomenon was first described by Lewis and Bloom (1972) at the species‐level, and as such, it was an early example of “speciation reversal” (see Seehausen et al., 2008). Since then, lineage fusion has been depicted for gene pools in general (i.e., including for incipient species and intraspecific clades) by Jansson and Dynesius (2002), Jesus et al. (2006), Rosenblum et al. (2012), and most recently, Dufresnes et al. (2023). Although lineage fusion can be a driver of extinction by introgression (Czekanski‐Moir & Rundell, 2019), it is also a creative force that might promote adaptive evolution and rapid radiation via the generation of a locally a high (albeit transient) excess of genetic diversity (Alcala & Vuilleumier, 2014), and the formation of novel heterozygous genotypes composed of deeply divergent alleles (“old” genetic variation) upon initial merging (Marques et al., 2019; Meier et al., 2023). Lineage fusion events are also important considerations in phylogeographic research, as the demographic history of a species may be affected by transient barriers, and inferences about the extent to which co‐distributed species had shared responses to past environmental change may be impacted by fission‐fusion (Garrick et al., 2019). That said, lineage fusion has rarely been reported at the intraspecific level (but see Garrick et al., 2014; Hinojosa et al., 2019; Kearns et al., 2018), perhaps owing to observation bias, as few researchers routinely consider or test for these events.

To obtain an appreciation of the prevalence of lineage fusion in nature, we need a better understanding of the circumstances under which these events produce recognizable genetic signatures and the potential for erroneously attributing these signatures to non‐fusion processes (Garrick et al., 2019, 2020). In phylogeographic studies that employ statistical hypothesis testing, the basic units of analysis are usually local population gene pools, since these permit use of coalescent modeling (Hey & Machado, 2003). Based on these units, empirical sample sizes, number of loci, DNA haplotype length, and observed levels of diversity are typically used to seed simulations conducted within the constraints of two or more competing models that differ in at least some parameter values (e.g., effective population sizes [*N*_e_], timing of events, etc.). Some approaches then use one or several summary statistics to characterize the simulated data, where the distribution of values represents expectations when a given model is true, and the same summary statistics calculated from the empirical dataset identify the best‐fit model (e.g., Bertorelle et al., 2010; see Garrick et al., 2019 for an example of ABC‐based hypothesis testing applied to lineage fusion). However, while some competing models are easily distinguishable, others can have partly overlapping summary statistic distributions, creating a zone of inference uncertainty (Nielsen & Beaumont, 2009). Indeed, if two or more models have fully overlapping summary statistic distributions, they are rendered untestable (Knowles, 2001, 2009).

To date, the extent to which lineage fusion produces distinguishable genetic signatures has been examined only for a limited set of non‐fusion demographic models. For example, Alcala et al. (2016) compared summary statistic values generated under fusion to those from population size constancy, extreme growth (i.e., 150‐fold increase in size in the past 2*N*_e_ generations), and a 3‐population Isolation with Migration model. Likewise, Garrick et al. (2019, 2020) compared lineage fusion to size constancy and expansion‐contraction. In the present paper, we consider the term “lineage” broadly to include entities at and below the species‐level, but we focus on intraspecific lineages (i.e., long‐isolated population gene pools) as the basis of simulations that were used to explore the distinguishability of lineage fusion against a much broader suite of non‐fusion scenarios, focusing on six commonly used demographic history summary statistics (i.e., neutrality tests). Rather than perform model selection or ranking (Garrick et al., 2019), the aim here was to expand on previous work that characterized the behavior of summary statistics (Garrick et al., 2020) but focusing now on the extent of overlap between the summary statistic values under the true lineage fusion scenario versus competing untrue scenarios. Given that some types of lineage fusion event may be less challenging to distinguish than others, simulations considered a range of different fusion scenarios. Give that population genetics and phylogeography of non‐model organisms have transitioned from data‐limited to data‐rich subdisciplines (Garrick et al., 2015), the potential for strategic allocation of DNA sequencing resources (i.e., number of individuals, loci, and base pairs [bp] sequenced per locus) to improve distinguishability was also examined.

## 2 METHODS

### 2.1 Basic approach

Pseudo‐observed datasets (PODs) were generated under four scenarios in which lineage fusion did occur. Next, the values of six demographic history summary statistics were estimated under the true lineage fusion scenario, as well as four major types of untrue non‐fusion scenario (i.e., constant size, growth, bottleneck, and decline). In all cases, summary statistics were estimated via coalescent simulations seeded using basic characteristics of the PODs. Finally, summary statistic distributions were characterized via their central 90% confidence interval (CI). As the basis for determining the circumstances whereby signatures of lineage fusion are identifiable, the central 90% CI of the true lineage fusion scenario was used as a benchmark against which the extent of overlap with the 90% CI of each untrue non‐fusion scenario was measured (detailed below). While this approach comes with the cavate of being unable to represent modality differences in summary statistic distributions, which may be informative (e.g., Alcala et al., 2016), it does facilitate direct comparison of several different compositions of simulated datasets (see below).

### 2.2 Simulation of pseudo‐observed datasets under lineage fusion

**FIGURE 1:**
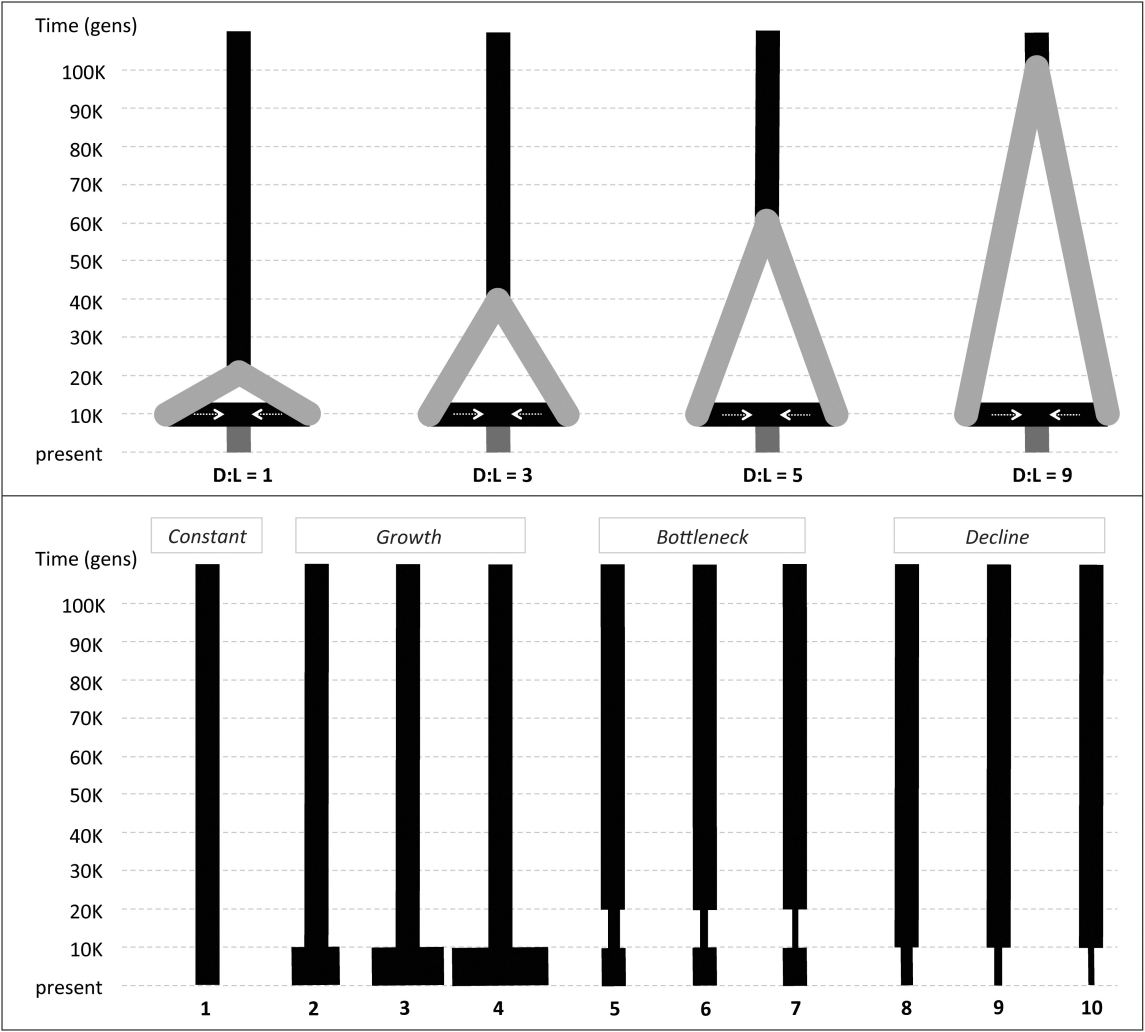
Graphical representation of population demographic models. Top panel: four lineage fusion scenarios with contrasting divergence time versus sampling lag time (D:L) ratios that were used to generate pseudo‐observed datasets (PODs), with base *N*_e_ = 10,000 and time in units of thousand (K) generations before present. Bottom panel: 10 untrue non‐fusion scenarios, representing four major classes of demographic model (constant size, growth, bottleneck, and decline) that were used to determine whether genetic signatures of lineage fusion are identifiable.

Following the approach of Garrick et al. (2019, 2020), simulations made the simplifying assumptions of drift‐induced divergence followed by instantaneous merging of two sister populations, and that only the present‐day panmictic gene pool could be sampled. PODs were simulated under four lineage fusion scenarios. While some of these reflect the timing of paleoclimatic events frequently cited in the literature (e.g., divergence originating during the penultimate glacial maximum or the Last Glacial Maximum, and merging occurring at beginning of the Holocene, assuming a 1‐year generation time), their key differentiating feature is the pre‐fusion divergence versus post‐fusion sampling lag time (D:L ratio herein), as this impacts detectability of fusion (Garrick et al., 2019). The four scenarios spanned a gradient of D:L ratios (i.e., 1, 3, 5, and 9), as these were indicated to lie with a “gray zone” where separation of true versus false demographic scenarios become challenging (Garrick et al., 2019). Parameters that defined these models were: (1) *N*_e_ (held constant at 10,000 diploid individuals along all branches of the population tree, such that fusion scenario emulated expansion‐contraction of suitable habitat); (2) timing of initial divergence (*t*_div_, either 20,000 40,000, 60,000 or 100,000 generations before present, depending on D:L ratio), (3) timing of subsequent fusion (*t*_fuse_, held constant at 10,000 generations before present); and (4) mixing (set at 0.5, such that each sister population contributed equally to a fusion event). All gene pool sampling occurred only in the present day (*t*_0_) (Figure 1, top panel).

The impact of allocation of sequencing resources upon the distinguishability of lineage fusion was also of interest. Accordingly, the number of sampled individuals, DNA sequence haplotype length, and number of diploid autosomal loci varied across PODs, but the total amount of sequence data per POD was held constant at 800,000 bp. As such, PODs contained either 5, 10, or 20 diploid individuals, 10, 50, or 100 phase‐known diploid loci, and locus length varied from 200 to 8000 bp. A total of nine different dataset compositions were simulated for under each lineage fusion scenario (Table 1). All PODs were simulated in DIY‐ABC v2.1.0 (Cornuet et al., 2014) using the HKY model of nucleotide sequence evolution (Hasegawa et al., 1985) with equal base frequencies, transition/transversion (ts/tv) ratio = 2, proportion of invariant sites = 10%, a gamma model for rate heterogeneity across sites (Yang, 1994) with two discrete categories, and a mutation rate (*μ*) of 1 × 10^−7^ substitutions per site per generation.

**TABLE 1:** Composition of pseudo‐observed datasets (PODs).

Alias	No. of alleles	No. of base pairs	No. of loci
POD‐1	10	800	100
POD‐2	10	1600	50
POD‐3	10	8000	10
POD‐4	20	400	100
POD‐5	20	800	50
POD‐6	20	4000	10
POD‐7	40	200	100
POD‐8	40	400	50
POD‐9	40	2000	10

*Note*: Partitioning of a fixed amount of DNA sequencing resources (800,000 base pairs per POD) varied along three axes: number of sampled alleles from the population gene pool (assuming a diploid out‐crossing species, two alleles per individual), number of base pairs per locus (i.e., haplotype alignment length), and number of bi‐parentally inherited, independent phase‐known autosomal loci.

### 2.3 Coalescent simulation‐based estimation of summary statistic values

There has been long‐standing interest in the use of neutrality tests for inferring population size changes (e.g., Excoffier et al., 2009; Ramírez‐Soriano et al., 2008 and references therein). Accordingly, this paper focused on six neutrality test summary statistics: Tajima's (1989) *D*, Fu and Li's (1993) F*, Fu's (1997) *F*_S_, Ramos‐Onsins and Rozas's (2002) *R*_2_, Achaz's (2008) Y*, and Kelly's (1997) *Z*_nS_ (Table 2). Summary statistic distributions were estimated via 1000 simulations using the “Coalescent Simulations (n‐loci|1‐pop)” feature in DnaSP v6.12.03 (Rozas et al., 2017), which implements routines from MLCOALSIM (Ramos‐Onsins & Mitchell‐Olds, 2007). Simulations were seeded using values for *θ* per gene (Watterson, 1975), number of sampled individuals, DNA sequence haplotype length, and number of diploid autosomal loci from the associated POD.

**TABLE 2:** Description of six summary statistics (neutrality tests) that were evaluated for their ability to detect unique signatures of lineage fusion, when compared to 10 untrue non‐fusion demographic scenarios.

Notation	Summary statistic	References	Basic description
*D*	Tajima's *D*	Tajima (1989)	Comparison of the mean number of pairwise nucleotide differences (*K*) versus the number of segregating sites (*S*), per locus
F*	Fu & Li's F*	Fu and Li (1993)	Comparison of the number of singletons (ƞ_S_) versus the mean number of pairwise nucleotide differences (*K*), per locus
*F*_S_	Fu's *F*_S_	Fu (1997)	Comparison of the observed number of haplotypes (*H*_N_) versus the expected number of haplotypes given the estimated value of *θ*_π_, per locus
*R*_2_	Ramos‐Onsins & Rozas's *R*_2_	Ramos‐Onsins and Rozas (2002)	Comparison of the number of singletons (*ƞ*_S_) versus the mean number of pairwise nucleotide differences (*K*), divided by the number of segregating sites (*S*), per locus
Y*	Achaz's Y*	Achaz (2008)	Comparison of the mean number of pairwise nucleotide differences ignoring singletons (*K* − *ƞ*_S_) versus the number of segregating sites ignoring singletons (*S* − ƞ_S_), per locus
*Z*_nS_	Kelly's *Z*_nS_	Kelly (1997)	A linkage disequilibrium‐based measure of allele frequency equivalency among pairs of segregating sites (*S*), within non‐recombining DNA regions

*Note*: Given that multi‐locus DNA haplotype datasets were simulated and analyzed, summary statistic values were averaged across loci.

Five types of demographic model were considered: lineage fusion, size constancy, growth, bottleneck, and decline. Parameters for the true lineage fusion scenario matched those used to generate the associated POD. A base *N*_e_ = 10,000 was used for all four untrue non‐fusion scenarios. Under the growth and decline models, timing of the demographic event was set at 1*N*_e_ generations before present (i.e., coinciding with the timing of merging under lineage fusion). To enable deeper insights into the circumstances under which lineage fusion becomes identifiable, three different magnitudes of growth (2×, 3×, or 4× base *N*_e_) and decline (0.5×, 0.33×, or 0.25× base *N*_e_) were modeled separately. Within the bottleneck models, size reduction was initiated at 2*N*_e_ generations before present and ended at 1*N*_e_ generations (i.e., recovery to original size coincided with the timing of merging under lineage fusion). Three bottleneck severities (0.5×, 0.33×, or 0.25× base *N*_e_) were modeled (Figure 1, bottom panel).

### 2.4 Distinguishability of lineage fusion

Outcomes from coalescent simulation‐based estimation of summary statistic values were summarized via 90% CIs. The proportion of the 90% CI of the lineage fusion scenario that was unique (i.e., not overlapped by a given non‐fusion scenario's 90% CI) was used to quantify distinguishability of lineage fusion. Here, several outcomes were possible. For example, the two distributions might be fully non‐overlapping in either direction (i.e., Fuse90_Lower_ > Nonfuse90_Upper_, or Nonfuse90_Lower_ > Fuse90_Upper_), in which case the proportion of the 90% CI of the lineage fusion scenario that was unique = 1. Alternatively, the two distributions might be only partly offset from one another. If the lineage fusion 90% CI was partly unique in the upper direction, this was calculated as the difference (Fuse90_Upper_ − Nonfuse90_Upper_) divided by the range (Fuse90_Upper_ − Fuse90_Lower_). However, if the offset was in the lower direction, the difference was calculated as (Nonfuse90_Lower_ − Fuse90_Lower_) divided by the range. Finally, if the non‐fusion scenario's 90% CI was completely nested within the lineage fusion scenario's broader 90% CI (i.e., there was partial offset in both directions), the upper and lower components were calculated as described above, then summed together. Ultimately, distinguishability of lineage fusion was assessed separately against each of 10 non‐fusion scenarios (i.e., size constancy and each of the three different magnitudes of growth, bottleneck, and decline).

### 2.5 Extended Bayesian skyline plot analysis of pseudo‐observed datasets

Assessment of the potential for genetic signatures of lineage fusion to resemble those of other demographic scenarios was expanded beyond neutrality test summary statistics by analyzing a subset of PODs using Extended Bayesian Skyline Plots (EBSPs; Heled & Drummond, 2008), implemented in BEAST v2.7.3 (Bouckaert et al., 2014). Given that demographic inferences from EBSPs are limited to size constancy, growth, decline, and combinations thereof (i.e., bottlenecks, etc.), yet the PODs were generated under lineage fusion, this assessment explored whether there were strong tendencies in the type of erroneous inference that might be drawn. For computational tractability, only PODs comprised of 50 loci (400 bp, 40 sampled alleles) were analyzed. Indeed, computationally intensive demographic analyses are often run with down‐sampled subsets of ~50 loci for non‐model species (Lado et al., 2020). Five replicate PODs for each of four lineage fusion scenarios (D:L ratios = 1, 3, 5 and 9) were run using the same model of nucleotide sequence evolution and mutation rate that generated the PODs, clock model = strict, operator weights = auto‐optimized, with other priors as default. Searches were conducted using 2.5 × 10^8^ Markov Chain Monte Carlo generations, sampling parameters every 5000th step, discarding 10% as burn‐in. Convergence for key demographic parameters was assessed using effective sample size values calculated in TRACER v.1.7.1 (Rambaut et al., 2018). If the 95% highest posterior density (HPD) of “number of population size changes” included 0, this was interpreted as failing to reject the null hypothesis of size constancy. If the 95% HPD excluded 0, then LOGCOMBINER and EBSPANALYSER were used to generate EBSP curves, and the direction(s) of size change was determined by visually assessing change in median *N*_e_ over time, relative to the 95% HPD of *N*_e_.

### 2.6 Summary statistic‐based demographic hypothesis testing

For comparison with EBSPs, the same set of 20 replicate PODs were examined for tendencies in the type of erroneous inference driven by failing to consider lineage fusion, using each of the six neutrality test summary statistics within a traditional hypothesis‐testing framework. Here, deviations from the null hypothesis of size constancy was evaluated by comparing observed summary statistic values against distributions simulated via neutral coalescence (1000 replicates) in DnaSP, with significance determined at the 0.05‐level (except for *F*_S_, where 0.02 was used following Fu, 1997). Growth was inferred from significantly negative *D*, F*, *F*_S_, or Y*, and from significantly small *R*_2_ or *Z*_nS_, whereas decline was inferred from significantly positive *D*, F*, *F*_S_, or Y*, and from significantly large *R*_2_ or *Z*_nS_. As for EBSPs, inferences were limited to a set of demographic histories that did not include the true history.

## 3 RESULTS AND DISCUSSION

### 3.1 Pseudo‐observed datasets

A summary of diversity within simulated DNA haplotype datasets is given in Table 3. Choices about the composition of PODs (i.e., individuals × number of loci × read length) were motivated by the desire to hold total number of base pairs per POD constant, so that optimal allocation of sequencing resources could be examined. As such, mimicking real datasets was not the primary goal. Nonetheless, most parameter values are generally representative of empirical datasets. For example, values for number of loci (10, 50, 100) are at the upper end of datasets produced via Sanger sequencing (Lee & Edwards, 2008), and the lower end of those generated using high‐throughput sequencing (Harvey et al., 2016). Values for locus length (200 to 8000 bp; Table 1) include those attainable from a several sequencing platforms. Unlike the simulated data used here, however, phasing of multi‐site heterozygotes in empirical datasets is often inferred computationally and therefore contains some error. That said, phasing algorithms generally perform well for phylogeographic datasets (Garrick et al., 2010), and phasing may not even be needed for some analyses (Robinson et al., 2014). Ultimately, the composition of the PODs was generally in‐line with empirical DNA sequence haplotype datasets, and as such, the associated inferences about distinguishability of lineage fusion should be broadly applicable. One potential exception to this relates to the overall level of polymorphism contained within PODs: averaged across all 52 PODs (Table 3), the mean per‐locus haplotype diversity (Nei, 1987) was *Hd* = 0.749 (range: 0.433–0.979), which may be considered somewhat higher than typically seen.

**TABLE 3:** Genetic diversity within pseudo‐observed datasets (PODs) simulated under four lineage fusion scenarios that differ in their duration of pre‐fusion divergence versus post‐fusion lag time prior to gene pool sampling (i.e., their D:L ratio; see Figure 1 top panel).

Composition of POD				Lineage fusion scenario			
Alias	No. of alleles	No. of base pairs	No. of loci	D:L = 1	D:L = 3	D:L = 5	D:L = 9
POD‐1	10	800	100	0.758 (0.035)	0.767 (0.040)	0.820 (0.029)	0.806 (0.043)
POD‐2	10	1600	50	0.844 (0.025)	0.874 (0.014)	0.884 (0.020)	0.910 (0.013)
POD‐3	10	8000	10	0.975 (0.001)	0.979 (0.001)	0.969 (0.003)	0.979 (0.001)
POD‐4	20	400	100	0.614 (0.054)	0.644 (0.054)	0.679 (0.047)	0.743 (0.040)
POD‐5	20	800	50	0.767 (0.029)	0.804 (0.021)	0.792 (0.027)	0.827 (0.028)
POD‐6	20	4000	10	0.954 (0.001)	0.945 (0.002)	0.964 (0.001)	0.949 (0.003)
POD‐7	40	200	100	0.433 (0.078)	0.489 (0.068)	0.539 (0.061)	0.555 (0.069)
POD‐8	40	400	50	0.581 (0.056)	0.650 (0.044)	0.679 (0.042)	0.724 (0.040)
POD‐8^b^	40	400	50	0.554 (0.068)	0.661 (0.039)	0.668 (0.050)	0.720 (0.049)
POD‐8^c^	40	400	50	0.571 (0.059)	0.620 (0.046)	0.709 (0.030)	0.739 (0.037)
POD‐8^d^	40	400	50	0.560 (0.064)	0.646 (0.043)	0.681 (0.040)	0.719 (0.048)
POD‐8^e^	40	400	50	0.574 (0.059)	0.657 (0.047)	0.721 (0.036)	0.703 (0.047)
POD‐9	40	2000	10	0.884 (0.005)	0.853 (0.015)	0.892 (0.009)	0.941 (0.001)

*Note*: Genetic diversity was summarized as haplotype diversity (i.e., the mean proportion of pairs of randomly selected haplotypes that are different from one another, averaged across loci; *Hd* (Nei, 1987)), with the sampling variance across loci (*Hd*_var_ (Nei, 1987)) given in parentheses. Superscripts identify replicate PODs that were generated to augment assessment of Extended Bayesian Skyline Plot outcomes.

### 3.2 Demographic history summary statistics, distinguishability of lineage fusion, and optimal allocation of sequencing resources

**FIGURE 2:**
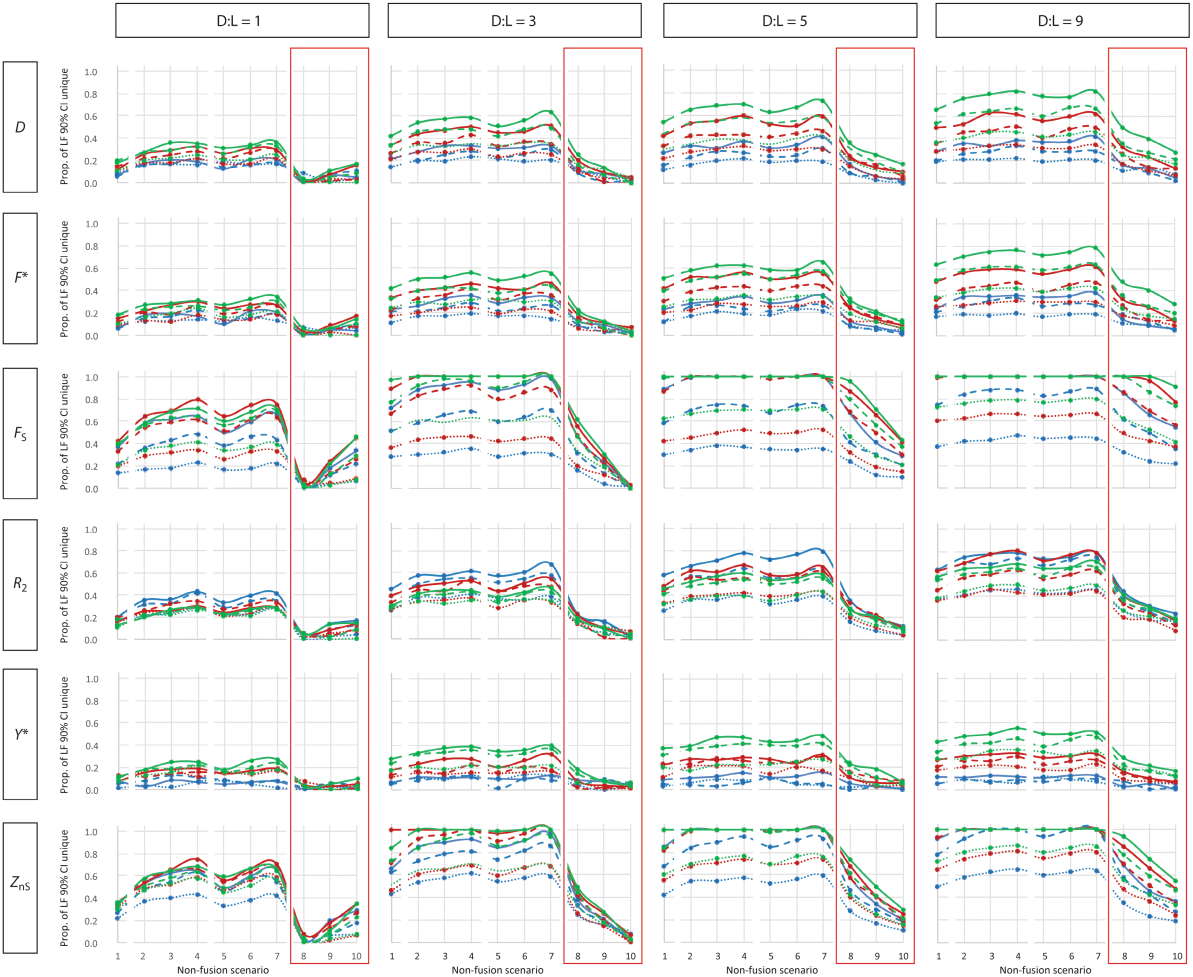
Distinguishability of lineage fusion and optimal sampling allocation. Four lineage fusion scenarios with contrasting divergence time versus sampling lag time (D:L) ratios are represented in columns, and six summary statistics are in rows. For each of the 24 plots, the *y*‐axis represents the proportion (Prop.) of the lineage fusion (LF) scenario's 90% confidence interval (CI) that is unique, compared to an untrue non‐fusion scenario's 90% CI. The *x*‐axis represents 10 untrue non‐fusion scenarios, which fall into four major classes (left to right: constant size [1], growth [2–4], bottleneck [5–7], and decline [8–10]; see Figure 1, bottom panel). Red boxes highlight the population decline scenarios. Symbol‐ and color‐coding of lines connecting data points on the plots indicate composition of the pseudo‐observed datasets (PODs) used to seed coalescent simulations, as follows: solid blue = POD‐1, dashed blue = POD‐2, dotted blue = POD‐3, solid red = POD‐4, dashed red = POD‐5, dotted red = POD‐6, solid green = POD‐7, dashed green = POD‐8, and dotted green = POD‐9 (Table 1). Note that these 10 categories are non‐continuous; line graphs within each major class are shown only to convey the symbol‐ and color‐coding information.

Of the six summary statistics considered here, *F*_S_ and *Z*_nS_ were most effective at capturing unique signatures of lineage fusion (i.e., they maximized the proportion of the 90% CI of the lineage fusion scenario that was not overlapped by a given non‐fusion scenario's 90% CI), particularly when PODs were simulated under scenarios with D:L ratios of 3 and above (Figure 2). Conversely, the lineage fusion scenario with D:L ratio = 1 had poor distinguishability regardless of which summary statistic was used. This is consistent with findings from earlier simulation studies showing that short periods of drift‐induced divergence prior to merging and/or long lag times between fusion and gene pool sampling, render these events difficult to detect (Garrick et al., 2019). Kearns et al. (2018) inferred lineage fusion in the Common Raven (*Corvus corax*), with initial lineage splitting estimated at ~1.5 million years ago, and subsequent reconnection occurring at least 140–440 thousand years ago, which corresponds with a D:L ratio of 3.4–10.7. Thus, depending on the circumstances, even “ancient” fusion events may be detectable, so long as the D:L ratio is favorable. As for why *F*_S_ and *Z*_nS_ were most effective, this may be related to their unique focus on whole DNA sequence haplotypes (cf. the other four statistics), which instead focus on features such as segregating sites, pairwise nucleotide differences, and/or singleton segregating sites (Table 2). Specifically, *F*_S_ uses information from the haplotype distribution, by comparing the observed versus expected number of haplotypes (Fu, 1997; Ramos‐Onsins & Rozas, 2002). *Z*_nS_ is a linkage disequilibrium‐based assessment of the pattern of associations among mutations at different polymorphic sites within a locus, and these patterns are interpreted in the context of haplotype frequencies within the population (Kelly, 1997). The *D*, F*, and *R*_2_ statistics performed similarly to one another, and were moderately capable of capturing unique signatures of lineage fusion depending on the D:L scenario, whereas the Y* statistic had very low power across all scenarios (Figure 2). Again, these findings are consistent with previous work (Garrick et al., 2020), but extend them to a broader array of untrue non‐fusion scenarios.

The most significant finding of this study was that signatures of lineage fusion closely resemble those of population decline (Figure 2). Except for the most favorable scenario (D:L = 9) coupled with the most efficient summary statistics (*F*_S_ or *Z*_nS_), the proportion of the 90% CI that was unique to lineage fusion was always quite low (<0.5). Furthermore, the CIs of the three population decline scenarios were occasionally completely nested within that of the lineage fusion 90% CI, such that a combination of lower and upper tails was used to calculate lineage fusion's distinguishability (this was most prevalent for *D*, F*, and Y*). From the perspective of statistical phylogeographic hypothesis testing (Knowles, 2001, 2009), this makes fusion versus decline particularly hard to distinguish (cf. a case where the offset between CIs is unidirectional). Another notable outcome was that while the magnitude of population decline was positively correlated with degree of difficulty in distinguishing it from lineage fusion in the less challenging scenarios (D:L ratios of 3, 5, and 9), this relationship reversed direction in the most challenging scenario (D:L = 1), suggesting that an inflection point lies around D:L = 2. The underlying reason for this reversal warrants further investigation, but the implication here is that the potential for ambiguous inference about demographic history would be greatest when a hypothesized lineage fusion event occurred relatively soon after initial divergence (e.g., a Last Glacial Maximum splitting event, followed by early Holocene reconnection, and merging), and a competing non‐fusion hypothesis includes population decline of only moderate severity (e.g., early Holocene contraction of habitat to approximately half of its former extent).

The reason why lineage fusion resembles population decline so closely is likely because they produce similar coalescent gene tree topologies. Both unrecognized population subdivision (which is a component of fusion) and population decline produce genealogies with long internal branches and an excess of old mutations (Ramírez‐Soriano et al., 2008). Ultimately, longer times are needed for all alleles sampled from a “population” to coalesce to their common ancestor. A pattern in which most pairwise differences among alleles are small, but some are very large, is precisely what is expected under both population subdivision and population decline, making the predictions of these hypotheses very hard to separate (Nielsen & Beaumont, 2009). That said, similar expectations also apply to population bottlenecks, and so the relative ease with which lineage fusion was distinguishable from each of the three bottleneck scenarios considered here is somewhat surprising. It may simply be that the magnitudes of reduction in *N*_e_ that were modeled in the bottleneck scenarios were all sufficiently strong such that lineages did not survive the bottleneck without coalescing (Ramírez‐Soriano et al., 2008), although this seems somewhat unlikely for the 0.5× bottleneck scenario. Timing of onset and recovery from the bottleneck may also be important parameters (Peery et al., 2012). Indeed, unlike the other non‐fusion scenarios where the major demographic event was initiated 1 *N*_e_ generations before present (coinciding with time of merging in the lineage fusion scenario), in the bottleneck scenarios, size reduction occurred 2 at *N*_e_ generations, with subsequent recovery at 1 *N*_e_ generations before present (Figure 1). This may mean that the decline phase of the bottleneck was too old to register long‐standing genetic signatures that mimicked fusion and/or the recovery phase was sufficiently recent that it did mimic signatures of the population growth scenarios that were generally quite distinguishable from lineage fusion. Ultimately, the impact of altering of bottleneck severity, timing, and perhaps also duration, warrants further investigation, but it beyond the scope of this study. Nonetheless, what is clear from the present work is that there is a close resemblance between signatures of fusion and decline, and ramifications of this are consequential. For instance, the inability to reliably infer lineage fusion can complicate conclusions about phylogeographic congruence among co‐distributed species (Garrick et al., 2019). Likewise, fusion events may play important roles in adaptive radiations (Marques et al., 2019), and affect inferences about macroevolutionary processes such as speciation and extinction rates (Alencar & Quental, 2021; Dynesius & Jansson, 2014; Rosenblum et al., 2012).

Given the potential importance of distinguishing lineage fusion from competing non‐fusion demographic hypotheses, an understanding of the optimal allocation of sequencing resources is valuable. For most of the summary statistics considered here, distinguishability of lineage fusion was highest when the dataset was composed of 100 loci with 20 or 40 sampled alleles (but 50 loci with 40 alleles also generally performed well). Conversely, 10 loci with 10 alleles almost always performed worst. Interestingly, *R*_2_ stood apart in that it seemed to benefit from longer haplotype lengths: 100 loci with only 10 alleles but 800 bp reads (POD‐1; Table 1) was the best or equally best performing dataset for this summary statistic (Figure 2). Few studies have investigated how the number of loci affects historical demographic inferences while holding the total the amount of sequence data constant. That said, Felsenstein (2006) considered the balance between haplotype length versus number of loci regarding accuracy of theta estimates for an isolated population, and clearly showed that it is better to it to add loci rather than extend read length. In the context of lineage fusion, Garrick et al. (2020) also explored impacts of haplotype length versus number of loci (again holding total the amount of sequence data constant) and reported that datasets with many short loci have an advantage over those with fewer long loci. Thus, findings from the present paper reaffirm the notion that maximizing the number of loci should be the priority. The additional axis of variation considered in this paper (i.e., number of diploid individuals) showed that whereas extending read length is rarely beneficial, more intensive sampling of individuals from a population can partly offset having fewer loci (Figure 2).

### 3.3 Potential for lineage fusion to mislead traditional demographic inferences

Coalescent‐based demographic reconstructions only provide biologically meaningful insights insofar as the pre‐defined set of models approximate the true history (Knowles, 2001, 2009). Many of these methods consider only size constancy, growth, and decline (e.g., Heled & Drummond, 2008; Hey, 2010), yet lineage fusion is fundamentally different given that it includes a distinct (albeit ephemeral) phase of divergence. Analysis of PODs using EBSPs, which do not consider lineage fusion, showed evidence of systematic bias in the resulting demographic inference (i.e., owing to model misspecification). Specifically, the null hypothesis of size constancy (which itself is untrue) was rejected in favor of population decline in 100% of the replicate PODs generated under the D:L = 5 and 9 lineage fusion scenarios. For the D:L = 3 scenario, this particular type of bias was much lower (20% of PODs), and non‐existent for D:L = 1 (Table 4). Interestingly, in some cases EBSPs seemed less prone to falsely inferring decline compared to most of the neutrality test summary statistics: for the D:L = 3 scenario, only *Z*_nS_ had an equally low bias in the direction of falsely inferring decline (Tables 4 and 5). Given these results, when analyzing empirical data and population decline is inferred using EBSPs or related methods, it may be prudent to explicitly include lineage fusion within the set of models considered in downstream analyses. For instance, Lado et al. (2020) inferred a severe population decline in dromedary camel ~700 thousand years ago using EBSPs, and Wei et al. (2020) concluded that some lineages of Jerdon's tree frog declined while others remained stable in response to Last Glacial Maximum climatic changes based on Stairway Plot analyses. These examples are mentioned not because there is an a priori expectation for lineage fusion, but rather, because both studies illustrate an opportunity to explicitly consider lineage fusion as an alternative explanation. Hinojosa et al. (2019) suggested that another potential indicator of a past lineage fusion could include deeply divergent yet broadly sympatric mitochondrial DNA lineages. Notwithstanding the possibility for this to arise by chance within a large panmictic population (Benham & Cheviron, 2019), such a pattern would indeed warrant careful examination, provided that the species is not already known to be a species complex. Additional criteria for lineage fusion candidacy might include the absence of spatial genetic structure or phylogeographic structure within a given species, despite its broad geographic distribution across an area known to have harbored multiple Pleistocene refugia, coupled with the presence of strong spatial genetic structure or phylogeographic structure of co‐distributed members of the same ecological community (Garrick et al., 2019). Other circumstances conducive to lineage fusion would include paleoclimatic ecological niche models and/or spatial projections of climatic stability surfaces (e.g., Garrick et al., 2021; Hyseni & Garrick, 2019) that clearly indicate historical disjunction of present‐day unstructured populations. Indeed, these and other criteria for lineage fusion candidacy could be used to formulate expectations prior to re‐analysis of publicly available genetic datasets from ecologically and taxonomically diverse taxa, to investigate the prevalence of lineage fusion in nature.

**TABLE 4:** Number of replicate pseudo‐observed datasets (PODs), out of five, for which population decline was falsely inferred (also differentially shaded) within a demographic hypothesis‐testing framework.

Basis of FALSELY Inferred decline	Simulated lineage fusion scenario			
	D:L = 1	D:L = 3	D:L = 5	D:L = 9
EBSP	0	1	5	5
*D*	0	3	5	5
F*	1	4	5	5
*F*_S_	0	3	5	5
*R*_2_	0	3	5	5
Y*	0	3	3	5
*Z*_nS_	0	1	5	5

*Note*: Extended Bayesian Skyline Plot (EBSP) and summary statistic (Table 2) approaches all assessed departure from the null hypothesis of size constancy, but none considered the true history of lineage fusion.

**TABLE 5:** Replicate pseudo‐observed datasets (PODs) for which departure from the null hypothesis of population size constancy was assessed (POD superscripts follow Table 3).

Basis of falsely inferred decline	Simulated lineage fusion scenario			
	D:L = 1	D:L = 3	D:L = 5	D:L = 9
Replicate 1: POD‐8				
EBSP	0 (0–2)	**3 (3–3)** ^**D**^	**1 (1–2)** ^**D**^	**1 (1–1)** ^**D**^
*D*	0.154	0.289*	0.314*	0.682*
F*	0.207*	0.189*	0.386*	0.695*
*F*_S_	0.191	1.080*	1.505*	3.296*
*R*_2_	0.124	**0.130** ^**L**^	**0.132** ^**L**^	**0.146** ^**L**^
Y*	0.044	0.282*	0.159*	0.380*
*Z*_nS_	0.172	**0.253** ^**L**^	**0.318** ^**L**^	**0.428** ^**L**^
Replicate 2: POD‐8^b^				
EBSP	0 (0–1)	1 (0–3)	**2 (1–3)** ^**D**^	**2 (2–4)** ^**D**^
*D*	−0.058	0.232*	0.261*	0.701*
F*	−0.046	0.224*	0.377*	0.609*
*F*_S_	−0.241	0.736*	1.667*	3.119*
*R*_2_	0.119	**0.126** ^**L**^	**0.127** ^**L**^	**0.145** ^**L**^
Y*	−0.093	0.187*	0.092	0.531 *
*Z*_nS_	0.169	0.210	**0.273** ^**L**^	**0.364** ^**L**^
Replicate 3: POD‐8^c^				
EBSP	1 (0–1)	0 (0–2)	**2 (2–4)** ^**GD**^	**2 (2–2)** ^**D**^
*D*	−0.181	0.072	0.668*	0.855*
F*	−0.258	0.214*	0.541*	0.777*
*F*_S_	−0.475	0.375	2.064*	3.139*
*R*_2_	0.111	0.123	**0.143** ^**L**^	**0.150** ^**L**^
Y*	−0.041	−0.058	0.555*	0.610*
*Z*_nS_	0.154	0.204	**0.310** ^**L**^	**0.381** ^**L**^
Replicate 4: POD‐8^d^				
EBSP	0 (0–1)	0 (0–3)	**3 (3–3)** ^**D**^	**3 (1–4)** ^**GD**^
*D*	−0.179	0.044	0.449 *	0.894 *
F*	−0.112	0.163	0.626 *	0.715 *
*F*_S_	−0.028	0.474	1.501 *	2.843 *
*R*_2_	0.117	0.120	**0.136** ^**L**^	**0.156** ^**L**^
Y*	−0.178	−0.059	0.135	0.735 *
*Z*_nS_	0.206	0.222	**0.269** ^**L**^	**0.350** ^**L**^
Replicate 5: POD‐8^e^				
EBSP	0 (0–1)	1 (0–3)	**1 (1–1)** ^**D**^	**2 (2–2)** ^**D**^
*D*	−0.083	0.367*	0.716*	0.354*
F*	0.008	0.319*	0.566*	0.398*
*F*_S_	0.427	1.043*	2.317*	2.204*
*R*_2_	0.126	**0.133** ^**L**^	**0.143** ^**L**^	**0.134** ^**L**^
Y*	0.149	0.248*	0.575*	0.205*
*Z*_nS_	0.233	0.235	**0.302** ^**L**^	**0.360** ^**L**^

*Note*: If the null hypothesis was rejected, values are bolded. For Extended Bayesian Skyline Plots (EBSP), the median “number of population size changes” (based on mean effective sample size = 119) is followed by the 95% highest posterior density in parentheses, and superscripts indicate the direction(s) of inferred population size change (^D^ = decline, ^GD^ = growth followed by decline). For the summary statistics *D*, F*, *F*_S_, and Y*, significantly positive values indicative of decline are marked with * (there were no significantly negative values). For *R*_2_ and *Z*_nS_, significantly large values indicative of decline are marked with ^L^ (there were no significantly small values).

## 4 CONCLUSIONS

Statistical phylogeography requires that competing hypotheses make quantitatively different predictions about patterns of genetic variation, and that the true demographic history is represented within the set. Over the past decade there has been increasing interest in understanding what types of unique genetic signatures arise when isolated and populations come into secondary contact and fully merge back together (e.g., Alcala et al., 2013, 2016; Alcala & Vuilleumier, 2014; Garrick et al., 2019, 2020). However, no study has assessed whether and when lineage fusion can be distinguished from decline. Here, simulations showed that this can be very difficult when pre‐fusion divergence time versus post‐fusion sampling lag time ratios are small. That said, if the circumstances of historical fusion and present‐day gene pool sampling are favorable (e.g., D:L ratio ≥ 3), and sensitive summary statistics such as *F*_S_ or *Z*_nS_ are used in combination with a relatively large number (i.e., 100+) of loci, then robust demographic inference may be possible. Follow‐up work is needed to explore the impact of simplifying assumptions made in construction of the demographic models (e.g., complete isolation during the divergence phase and instantaneous merging for the lineage fusion scenario, instantaneous growth, and/or decline for the non‐fusion scenarios) and the simulated genetic datasets used to assess them (e.g., recombination‐free, independent phase‐known DNA haplotype loci). In the meantime, a broader awareness of the potential for lineage fusion to carry the hallmarks of population decline is urgently needed.

## AUTHOR CONTRIBUTIONS

**Ryan C. Garrick:** Conceptualization (lead); data curation (lead); formal analysis (lead); funding acquisition (lead); investigation (lead); methodology (lead).

## CONFLICT OF INTEREST STATEMENT

The author declares no conflict of interest.

## Data Availability

Simulated DNA sequence haplotype datasets and their estimated summary statistic distributions are available via Dryad Repository entry https://doi.org/10.5061/dryad.1jwstqk11.
